# Broadcast Propagation Time in SpaceFibre Networks with Various Types of Spatial Redundancy

**DOI:** 10.3390/s23136161

**Published:** 2023-07-05

**Authors:** Valentin Olenev, Elena Suvorova, Nadezhda Chumakova

**Affiliations:** Aerospace R&D Centre, Saint-Petersburg State University of Aerospace Instrumentation (SUAI), 190000 Saint-Petersburg, Russia; wildcat15@yandex.ru (E.S.); nadezhda.chumakova@guap.ru (N.C.)

**Keywords:** SpaceFibre, broadcast, fault mitigation, FDIR, Petri nets, timed automata

## Abstract

Various methods of spatial redundancy can be used in local networks based on the SpaceFibre standard for fault mitigation of network hardware and physical communication channels. Usually, a network developer chooses the method of spatial redundancy according to the number of failures that have to be mitigated, the time required for restoring the normal operation of the network, required overheads and hardware costs. The use of different spatial redundancy mechanisms can cause changes in the structure of the links between network nodes, in case of failure and subsequent mitigation. In turn, this may cause changes in the broadcast transmission paths and the temporal characteristics of their delivery from the source to the receivers. This article focuses on the change in the propagation time of broadcasts in SpaceFibre networks with spatial redundancy. Broadcast propagation rules significantly differ from data-packet propagation rules. Broadcast distribution time is very important for many applications, because broadcasts are generally used to send urgent messages, in particular for time synchronization. Various formal methods have been used to evaluate the propagation characteristics of the broadcast. A method for estimating broadcast propagation time along the shortest routes is proposed. In addition, we provide a formal method to estimate the number of failures, which occurred in the network during the broadcast propagation. This method is based on timed Petri nets; one of its features is the ability to calculate broadcast transmission delays. In addition, as an alternative solution, we propose a method for estimating delays based on time automata theory.

## 1. Introduction

Most local area networks (LANs) require mechanisms to mitigate failures and faults. The SpaceFibre standard [1] specifies methods for fault detection, isolation and recovery, but does not specify particular mechanisms for fault mitigation and methods of spatial redundancy. Currently, LANs use various methods of spatial redundancy. They differ in connection schemes for redundant components (components can be in hot or cold redundancy) and in the rules for building of a linked graph of the network (network structure).

Each type of spatial redundancy has different achievable network characteristics, such as the amount of additional equipment used, additional power consumption, normal operation recovery time and network tolerance characteristics. None of the existing methods is suitable for any type of network. Developers choose the most appropriate method according to the system tasks and the acceptable overhead costs.

The choice of the most suitable spatial redundancy method is based on the transmission characteristics and distribution rules of various types of data under conditions of different kinds of failure. Note that the broadcast propagation rules are significantly different from the rules used for data packets. There are a large number of publications in which data packet propagation time for SpaceFibre networks with fault mitigation was estimated. Such evaluations have not been made before for broadcasts. In particular, the transmission characteristics of broadcast messages can be very important for many applications, since they are used to transmit highly important information. In this article, we evaluate the dependence of the broadcast propagation time from the chosen spatial redundancy scheme under network failure conditions. As a result, the effect of the used spatial redundancy method on their propagation time will be different than for data packets.

A new method based on the formal technique of Petri nets is proposed to estimate the mitigation of the number of faults during broadcast transmission. Using this method, it is possible to convert the broadcast propagation through the SpaceFibre network into a timed Petri net. By analyzing the reachability of the resulting Petri net, it is possible to evaluate the effectiveness of the chosen redundancy method and the number of failures that the network can mitigate. In addition, the time component of the Petri net, embedded in the method, allows calculating the delay of broadcast transmission to various network nodes.

To verify and analyze the characteristics of broadcast transmission in SpaceFibre networks, taking into account the dynamics of failures and faults, the formal technique of timed automata is used. This technique was developed for verification and analysis of the characteristics of systems with real-time requirements [2,3,4]. Special variables (timers or clocks) are used to add the notion of time to formal models [5,6]. Timed automata could also include variables of other types. For example, these variables can be used to count events [7,8,9,10]. Timed automata use a special additional list of parameters which are represented in detail in [8,9,11,12]. To study complex systems, networks of timed automata can be built [13,14,15,16]. Interaction between automata is carried out through channels or through the use of common variables [7,8,15,16]. Events (signals) can be transmitted through channels. Each channel has one source and can have one or more receivers. Channels can be implemented as synchronous or asynchronous. In the first case, the transition that puts the event into the channel fires only if the transition that reads the event from the channel can (and does) fire. Such transitions can be used to synchronize individual timed automata in the network. If the transition is asynchronous, the reading of the event from the channel can be delayed in relation to the moment when the event is written to the channel [15,16,17,18]. In this paper, we propose an approach based on the networks built upon the timed automata to dynamically analyze broadcast transmission characteristics.

## 2. Broadcast Message Propagation Rules for SpaceFibre Networks

A broadcast message is transmitted in the SpaceFibre network from one source to all network nodes (routers and terminal nodes). A particular network node can process or discard a broadcast depending on its type (receive only specific types of broadcasts). A broadcast propagation mechanism prevents the message looping in the networks, whose structure contains link cycles. This mechanism is based on the use of a dynamically generated broadcast distribution tree. The following actions are performed in routers upon receiving of the next broadcast. If such a broadcast is received in the router for the first time since the start of operation, then it is sent to all ports of the router, except the one which it came from. The number of the port from which the broadcast was received should be saved, and then a timeout timer should be started. The timeout duration has to be longer than the broadcast propagation time over the longest cycle in the network. If a broadcast of this type has already arrived at the router, it is checked which port it previously came from. If the broadcast came from the same port, it should be sent further. Otherwise, the expiration of the timeout should be checked. If the timeout has not expired, then the broadcast is identified as duplicate and discarded. If the timeout has expired, the broadcast, which is propagated through the network in a different way, is considered to be a new one, because the network configuration has changed. This could happen because of a network fault. The result of the operation is building of a broadcast propagation tree. This tree can be dynamically rebuilt if the network fails. Broadcast propagation time to various network nodes may change when rebuilding the tree.

If the source sends a broadcast of the same type with the same channel ID more often than the timeout duration, then the broadcast might be lost during the rebuilding of the propagation tree (not delivered to some network nodes).

## 3. Evaluation of Broadcast Propagation Time in SpaceFibre Networks

The maximum broadcast transmission time from the source node to different devices in SpaceFibre networks is different and depends on the length of the broadcast transmission route. In this article, we use the Lee algorithm to calculate the lengths of the shortest routes [19,20].

In addition, the maximum broadcast transmission time depends on the processing time in transit switches located on the route and the receiving device, on the transit input and output port delays of all devices on the route, and on the broadcast transit time through physical channels. These parameters are already known at the network design stage.

Since the paths of broadcast can change due to the dynamic change of the broadcast distribution tree, the maximum broadcast transmission time can also be changed.

Article [21] provides an equation and formal scheme for evaluation of the broadcast distribution time via a router. However, this calculation scheme is focused on several specific devices implemented in the FPGA, which are discussed in that article. We offer a generalized calculation scheme, focused on a wider class of devices. In this scheme, delays are divided into the following main groups: delays in the input port, delays in the output port, delays at the network level, and delays in the communication channel between neighboring devices. The values of these delays can be determined based on the analysis of the RTL (Register Transfer Level) models (written in VHDL or Verilog), which are clock-accurate models of real circuits. These values depend on the specifics of the device implementation, such as:

− sizes of elastic buffers,
− width of the broadcast transmission channels inside the devices (between separate units),
− periods of clocks,
− number of cycles spent on performing various actions,
− service buffers and registers (which may be required to eliminate long communication lines between individual units) and other specific components determined by the implementation technology and the technology libraries used.

During the analysis of the RTL model, it is necessary to determine the areas in which delays are strictly deterministic (depending only on the period of the corresponding clock signal) and areas in which delays can be changed. 

The reasons for the variability (the sources of the variability) are determined for areas where delays may fluctuate. The main reasons include a non-deterministic phase shift between clocks, a discrepancy in the clock periods and competition for resources (for example, waiting for the previous symbol to be sent). Note that additional reasons may exist for particular implementations.

If several variations are found, it is necessary to determine how they contribute to the transmission time increasing in relation to each other. In some cases, they can “extinguish” each other. This happens, for example, if they wait for two different events overlapping in time (completely or partially). In other cases, they may summarize. This happens, for example, when data objects of different types can compete at the input to the buffer, and the write and read clocks for the buffer can be shifted arbitrarily. In some cases, delays can be multiplied. This can happen, for example, if the data object consists of several atomic elements. For each atomic element, there is a resource contention phase and transmission through the time domain phase (clocks may be out of phase and not coincide in period), and the resource contention phase cannot coincide in time with the write phase for the previous atomic element. Further, according to these factors, the maximum data transmission time is evaluated.

The maximum broadcast transmission time for a device is calculated by the following equation:

(1) $$\lambda t\max=\sum_{j=1}^{N-1}(\lambda port\_out_{j})+\sum_{j=2}^{N}(\lambda port\_in_{j})+\sum_{j=2}^{N}(\lambda n_{j})+\sum_{j=1}^{N-1}(\lambda phy_{j})$$

where:

- N is the number of devices on the broadcast transmission route, including the source node and the destination node;
- λport_out_j_ is the delay in the broadcast passing through the output port of device j;
- λport_in_j_ is the delay in the broadcast passing through the input port of device j;
- λn_j_ is the broadcast processing time in device j;
- λphy_j_ is a channel connecting device j with device j + 1.

All devices on the route are numbered from 1 (source node) to N (destination node) to simplify the equation.

**Figure 1:**
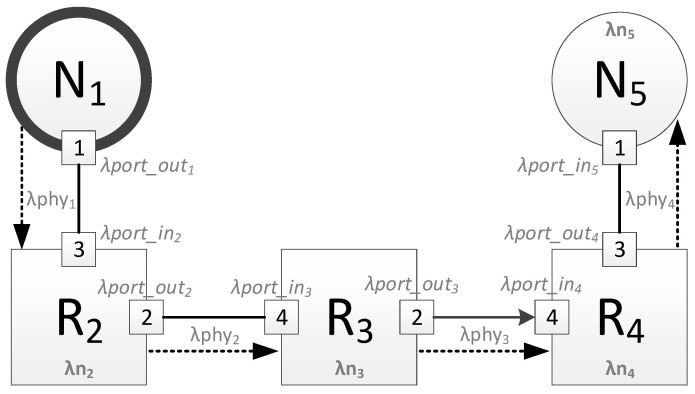
Example of a broadcast route.

The Figure 1 shows an example of a route through five devices. For this route, the maximum broadcast transmission time will be calculated using the following equation:

(2) $$\lambda t\max=\sum_{j=1}^{4}(\lambda port\_out_{j})+\sum_{j=2}^{5}(\lambda port\_in_{j})+\sum_{j=2}^{5}(\lambda n_{j})+\sum_{j=1}^{4}(\lambda phy_{j})$$

## 4. Estimation of Broadcast Propagation Time Change 

### 4.1. Considered Methods of Spatial Redundancy

The most widely used in LANs methods of spatial redundancy are represented in this section [22,23].

The first method uses K + 1 identical networks (in case of mitigation of K faults). These networks are not interconnected, but each terminal node is connected to all networks. In particular, this approach is used within the framework of the AFDX standard [24,25,26].

The second method uses a single network which includes both the main and redundant routers. In some cases, each router has its role—main or redundant; in other cases, there is no division. For this method, the rules for adding redundant routers, the number of added routers, and redundant and main router linking rules can significantly vary. One of the most popular implementations of this method is making K + 1 identical networks (like in first method), but there are “cross” links between routers from different networks [22,23].

Both of the above spatial redundancy methods can be applied to SpaceFibre networks.

### 4.2. Fault Mitigation by Using Identical Networks

There are K + 1 completely identical, not interconnected, SpaceFibre networks. A separate broadcast distribution tree is built in each of these networks. Broadcast distribution trees may not be identical. This happens when there are multiple broadcast paths in the network with relatively equal characteristics. The broadcast maximum transmission time to any i terminal node over all K + 1 trees is also relatively equal.

If one of the networks fails, it will rebuild the broadcast propagation tree for that network, but it will not affect the broadcast propagation trees for other networks. As a result, if at most K failures occur, then at least one broadcast propagation tree will remain unchanged. Thus, the maximum transmission time of the broadcast will not change. Also, it will not change for those broadcasts that were transmitted directly during the network failure.

#### 4.2.1. Identical Networks for Networks with Tree Topology

**Figure 2:**
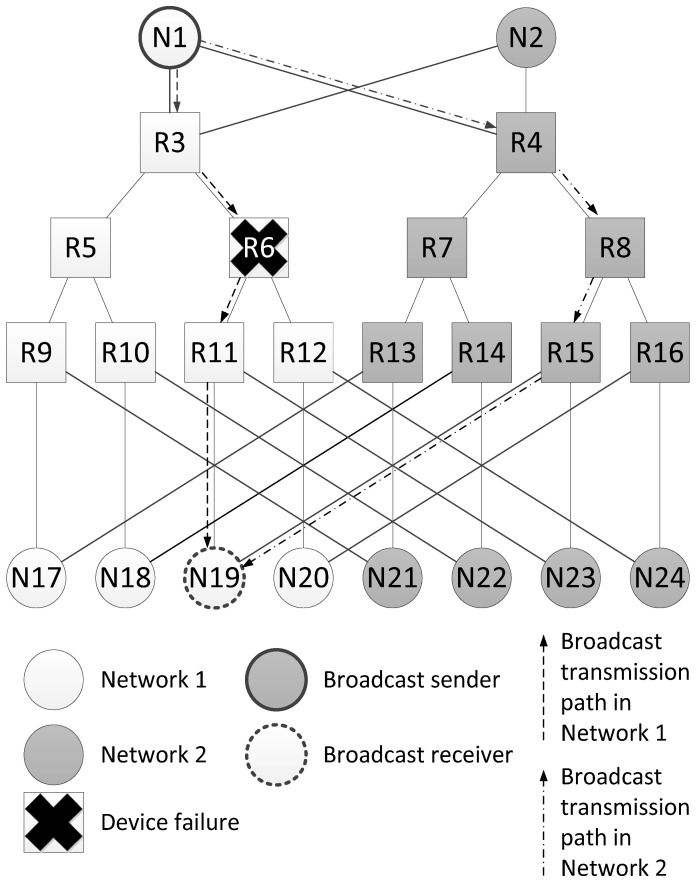
An example of fault mitigation in a network with a tree topology.

The Figure 2 shows an example of this spatial redundancy method. The example uses two identical networks with a tree topology. The terminal nodes of these networks are connected to both networks. Such a structure allows mitigating one fault.

Let us consider an example of fault mitigation using this spatial redundancy method for the network shown in Figure 2. It shows two broadcast propagation routes from the broadcast packet source N1 to the node N19. The first route passes through Network 1 (routers R3, R6 and R11), and the second one passes through Network 2 (routers R4, R8 and R15). Both routes are four hops long. When the network operation is correct, node N19 receives two identical broadcast packets from source N1. In the example shown in Figure 2, router R6 failed. Broadcast is transmitted along two routes, but node N19 receives the broadcast only once.

In this example, the failure of router R6 makes the transmission of broadcasts along routes passing through routers R11 and R12 and connected to router R6 impossible. However, using identical networks allows sending broadcasts over alternative routes of the same length as the original ones. Moreover, despite a router failure, all terminal nodes will receive at least one broadcast.

**Figure 3:**
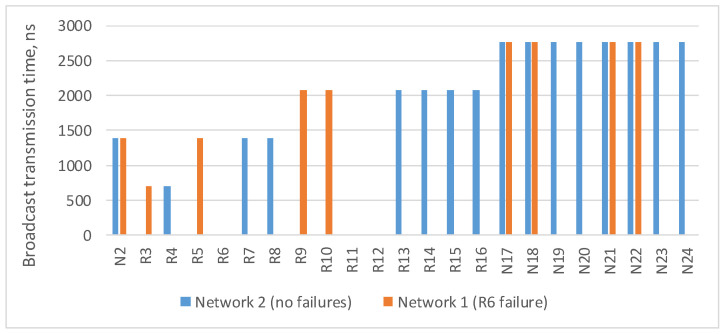
Broadcast maximum transmission time in a network without failures and in a network with a failure of router R6.

The Figure 3 shows a histogram with estimation of the maximum broadcast transmission time from the source node N1 to all devices in the network. This histogram shows that routers R6, R11 and R12 did not receive a broadcast, and nodes N19, N20, N23 and N24 connected to them received only one broadcast. The remaining terminal nodes will receive two broadcasts, and the broadcast maximum transmission time for both routes is the same.

**Figure 4:**
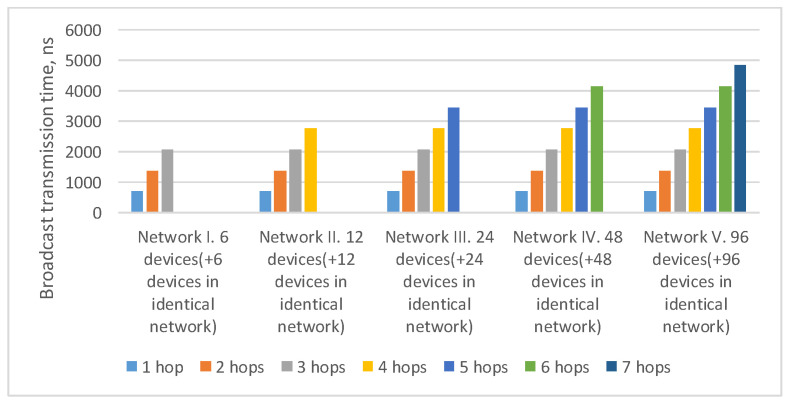
Broadcast maximum transmission time in networks with a tree topology with different route lengths (spatial redundancy with identical networks).

In the current example, the maximum route length is four hops: from the broadcast source node N1 to nodes N17–N24. As the number of devices in the network increases/decreases, the maximum route length can also increase/decrease. The histogram in Figure 4 shows a broadcast maximum transmission time over networks with a tree topology with different route lengths. This histogram shows that the maximum transmission time for routes of the same length in all networks is the same. The maximum transmission time increases linearly with the maximum route length.

#### 4.2.2. Identical Networks for Networks with 2D-Grid Topology

Similarly, fault mitigation is performed on other topologies. Let us consider an example of fault mitigation for a network with a two-dimensional grid topology.

**Figure 5:**
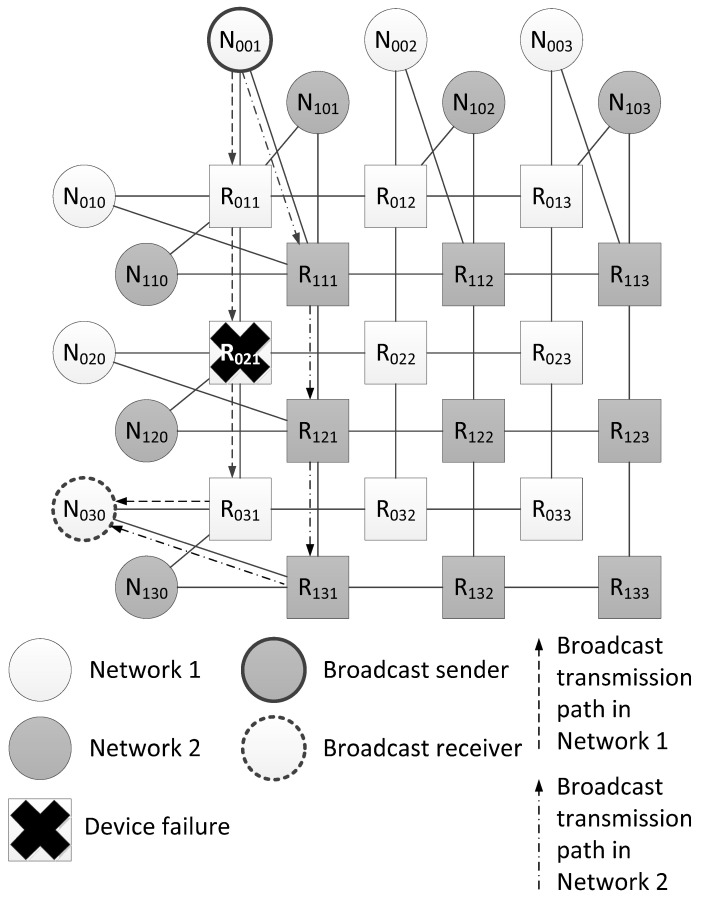
Example of fault mitigation in a network with a 2D-grid topology.

An example from Figure 5 shows two broadcast propagation routes over the network from the source N001 to the node N030. The first route passes through Network 1 (routers R011, R021 and R031), and the second one passes through Network 2 (routers R111, R121 and R131). Both routes are four hops long. When the network operation is correct, node N030 receives two identical broadcast packets from source N001. In the example shown in Figure 5, router R021 failed. Due to the fact that the broadcast is transmitted along two routes, node N030 receives only one broadcast.

In contrast to the previously considered network with a tree topology, in a network with a 2D-grid topology, the failure of router R021 does not make it impossible to send a broadcast through other network routers. However, in this example, the length of the broadcast route from the source node R001 to the router R031 has increased by two hops.

**Figure 6:**
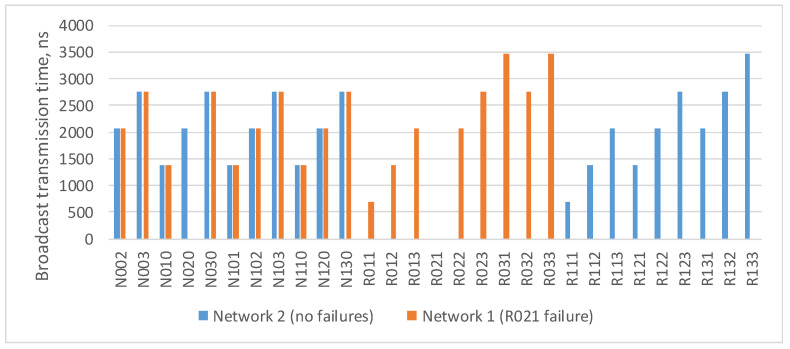
Broadcast maximum transmission time in a network without failures and in a network with a failure of router R021.

Figure 6 shows a histogram of the broadcast maximum transmission time from the source node N001 to all devices in the network. This histogram shows that router R021 did not receive a broadcast, and node N020, connected with it, received only one broadcast. The remaining terminal nodes received both broadcast packets, and the broadcast maximum transmission time for the two routes will be the same.

**Figure 7:**
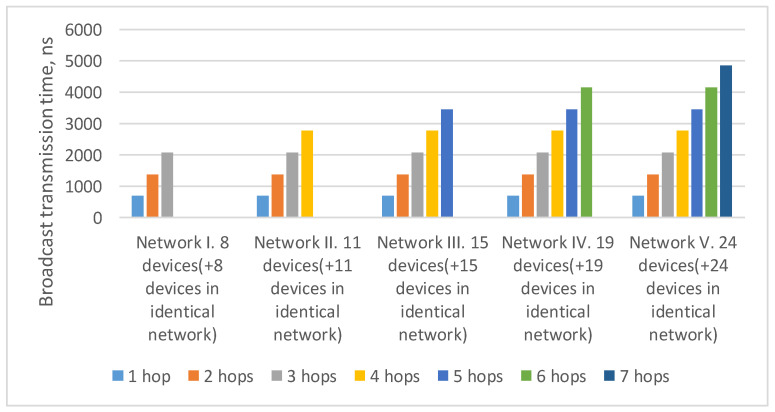
Broadcast maximum transmission time in networks with a 2D-grid topology with different route lengths (spatial redundancy with identical networks).

In current example, the maximum route length is five hops: from the broadcast source node (N001) to nodes N033 and N133. As the number of devices in the network increases/decreases, the maximum route length can also increase/decrease. Histogram on the Figure 7 shows a broadcast maximum transmission time over networks with a 2D-grid topology with different route lengths. This histogram shows that the maximum transmission time for routes of the same length in all networks is similar. At the same time, the maximum transmission time increases linearly with the maximum route length.

If there are cycles in networks that use this spatial redundancy method, the presence of independent propagation trees provides the ability to send broadcasts of the same type more often than the maximum transmission duration per cycle (maximum timeout time). Such broadcasts will be delivered to network terminal nodes along the trees that have not changed.

### 4.3. Fault Mitigation Using Redundant Routers and Cross-Links 

A single broadcast distribution tree is built for this spatial redundancy method. It could include both primary and redundant routers (except the cold redundant routers). If any of the routers or communication channels included in the distribution tree fails, the broadcast distribution tree dynamically changes.

In this case, it is potentially possible that the transmission time over the new tree will be close to the transmission time over the original tree. 

#### 4.3.1. Redundant Routers in Networks with 2D-Grid Topology

**Figure 8:**
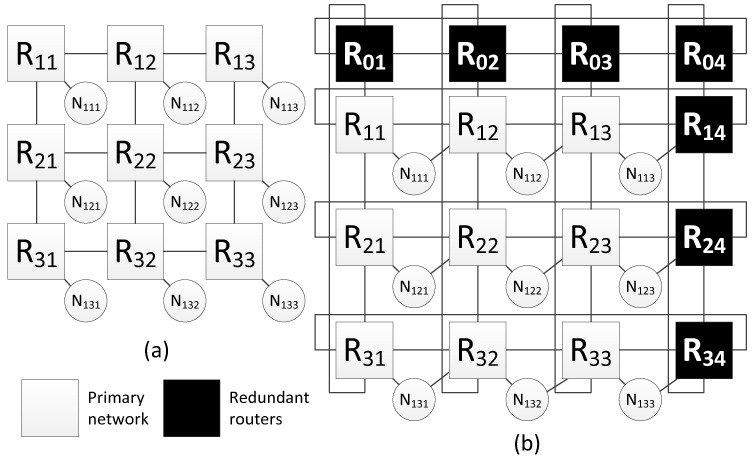
Network with a 2D-grid topology: (**a**) without redundant routers, (**b**) with redundant routers.

The Figure 8a shows an example of a network with 2D-grid topology, which current method of spatial redundancy is used for. In Figure 8b, redundant routers are included in this network in such a way that an additional row and column is added to the two-dimensional grid. After adding redundant routers, each row and column is closed, transforming into a torus topology. Also, for each terminal node, an additional link is added with a neighbor router (located in the same row).

**Figure 9:**
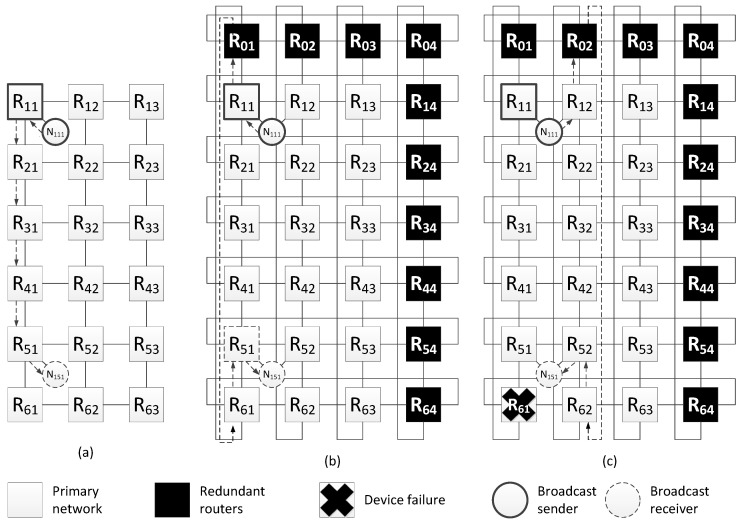
Example of fault mitigation in a network with a 2D-grid topology: (**a**) original route; (**b**) route through the redundant router; (**c**) route through the redundant router with router R61 failure.

Let us consider an example of fault mitigation for a 2D-grid topology using this spatial redundancy method. Figure 9a shows a network with a two-dimensional grid topology and one of the broadcast transmission routes. In this network, a terminal node is connected to each router; however, the figure shows a connection scheme for only two terminal nodes (N111 and N151), and the rest of routers are connected in a similar way.

This example shows one of the broadcast transmission routes. The broadcast source is node N111 connected to router R11, and the receiver is node N121 connected to router R51. The length of the original broadcast transmission path is six hops. Including redundant routers has made it possible to shorten the route; the new route is five hops (see Figure 9b). 

During the transmission of a packet from the source to node N151 (connected to router R51), the R61 or part of it failed. In this case, the route will be changed. The modified route is shown in Figure 9c; it is also five hops long.

**Figure 10:**
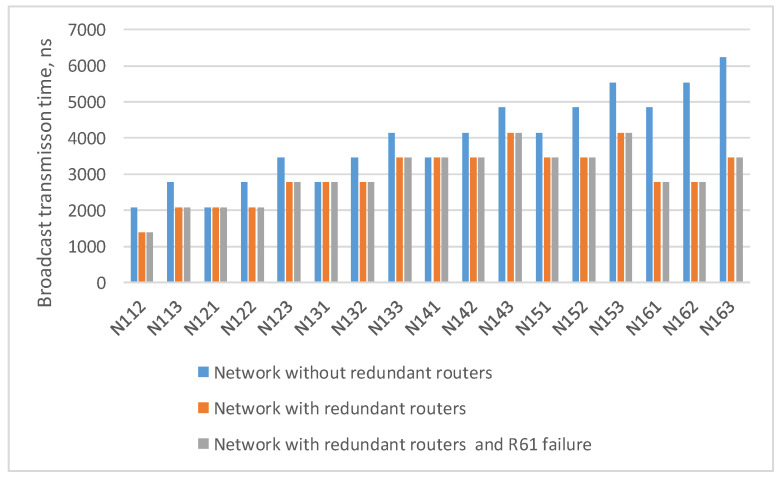
Broadcast maximum transmission time from node N111 to all devices in a network without redundant routers, in a network with redundant routers, and in a network with failure of router R61.

Histogram from the Figure 10 shows that when redundant routers are added, the broadcast maximum transmission time from the source node N111 either remains the same (for terminal nodes N121, N131, N141) or decreases, and the failure of router R61 does not affect the broadcast transmission time.

#### 4.3.2. Calculation of Transmission Route Length in Networks with 2D-Grid Topology

Adding redundant routers and cross-links can help to shorten the broadcast transmission route. For the case when the broadcast sender and receiver are connected to routers located in the same column, the length of the alternative route L_col_ is calculated by the equation:

(3) $$L_{col}=N+2-H$$

where H is the length of the original route in hops, and N is the number of devices in the column (taking into account the redundant router added to the column).

If the length of the alternative route is less than the length of the original route (L_col_ < H), broadcasts will be transmitted along the alternative route through the redundant router.

For the case when the broadcast sender and receiver are connected to routers located on the same row, the length of the original route is reduced by 1 hop:

(4) $$H^{\prime }=H-1$$

The reduction of the original route is performed by adding links of terminal nodes with neighbor routers located in the same line. In this case, the length of the alternative route is calculated by the following equation:

(5) $$L_{row}=N+2-H^{\prime }$$

where H is the length of the original route in hops, and N is the number of devices in the row (including the redundant router added to the row). If the length of the alternative route is less than the length of the original route (L_row_ < H′), broadcasts will be transmitted along the alternative route through the redundant router.

Let us calculate the length of the alternative route for the case when the broadcast sender and receiver are connected to routers located in different rows and columns. In this case, the length of the original route is divided into two components: the route in the column (H_col_) and the route in the row (H_row_):

(6) $$H=H_{col}+H_{row}$$

The length of the original route should be reduced by 1 hop (H′ = H − 1), by reducing the length of the route in row by 1 hop (H′_row_ = H_row_ − 1):

(7) $$H^{\prime }=H_{col}+H_{row}^{\prime }$$

In this case, the length of the alternative route is calculated by the equation:

(8) $$L_{row\_col}=(N_{col}+2-H_{col})+(N_{row}+2-H_{str}^{\prime })$$

where N_col_ is the number of devices in the column (including the redundant router added to the column), and N_row_ is the number of devices in the row (including the redundant router added to the row).

If the length of the alternative route is less than the length of the original route (L_row_col_ < H′), broadcasts will be sent along the alternative route through the redundant router.

**Figure 11:**
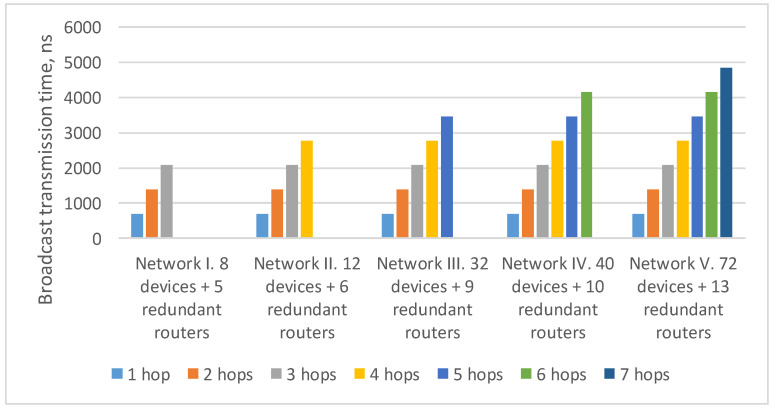
Broadcast maximum transmission time in networks with a 2D-grid topology with different route lengths (spatial redundancy with redundant routers).

In the previously considered example (Figure 9), the maximum route length is six hops: from the broadcast source node N111 to node N153. As the number of devices in the network increases/decreases, the maximum route length can also increase/decrease. Histogram on the Figure 11 shows the transmission time of the broadcast over networks with a 2D-grid topology with different route lengths. The histogram shows that the transmission time for routes of the same length in all networks is the same. At the same time, the maximum transmission time increases linearly with the maximum route length.

#### 4.3.3. Redundant Routers in Networks with Tree Topology

Let us consider an example of fault mitigation for a network with a tree topology.

**Figure 12:**
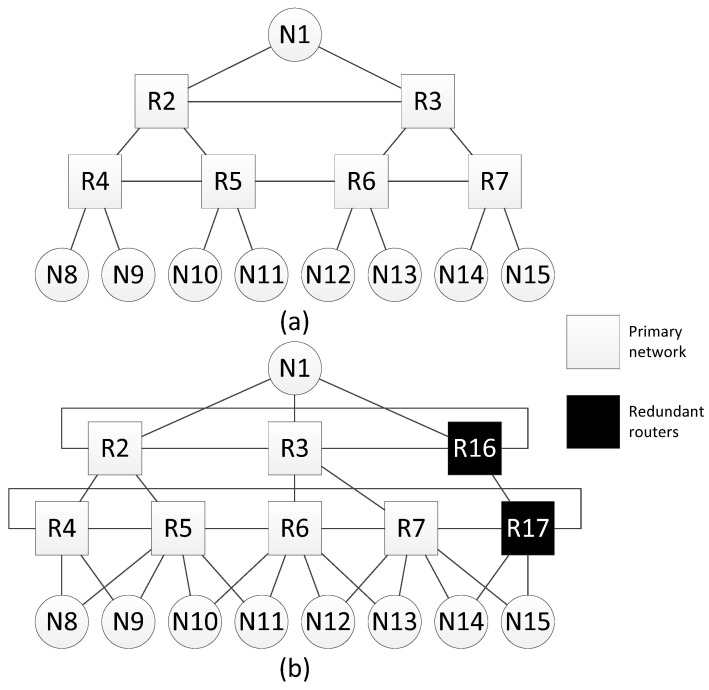
Network with a tree topology: (**a**) without redundant routers, (**b**) with redundant routers.

The Figure 12a shows an example of a network with a tree topology, for which this method of spatial redundancy is used. In Figure 12b, redundant routers are included in each line of routers of this network. After adding redundant routers, each line of routers is closed, transforming into a torus topology. In addition, for each terminal node, an additional link with a neighbor router (located on the same line) is added. 

**Figure 13:**
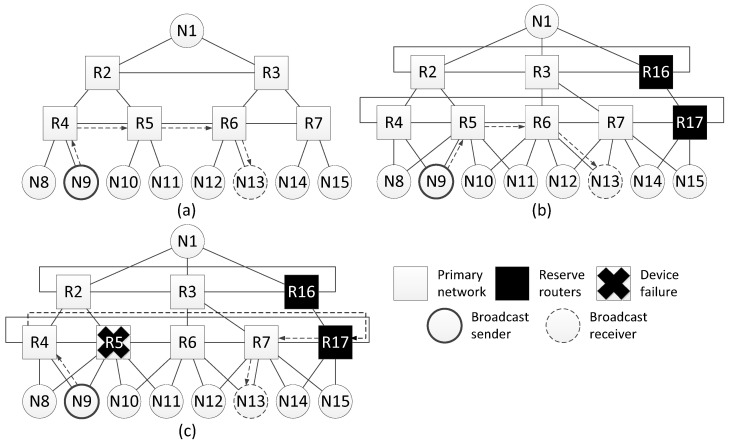
Example of fault mitigation in a network with a tree topology: (**a**) original route; (**b**) route through the redundant router; (**c**) route through the redundant router with failure of router R5.

Consider an example of fault mitigation for a tree topology using this spatial redundancy method. The Figure 13a shows a network with a tree topology and one of broadcast transmission routes. The sender of the broadcast is node N9, the receiver is node N13. The original broadcast transmission path is four hops long. Adding redundant routers and cross-links makes it possible to shorten the route, and the new route is three hops long (Figure 13b).

Let us assume that during the transmission of a packet from the source to node N9, router R5, or part of it, fails. In this case, the route will be changed. The modified route is shown in Figure 13c; it is also five hops long.

**Figure 14:**
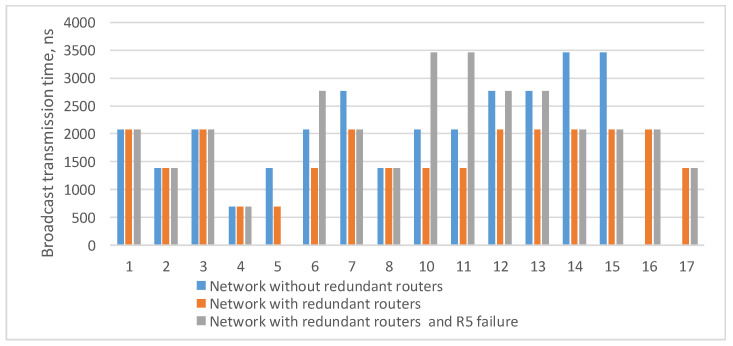
Broadcast maximum transmission time from node N9 to all devices in a network without redundant routers, in a network with redundant routers, and in a network with failure of router R15.

Histogram on the Figure 14 shows that when redundant routers are added, the broadcast maximum transmission time remains the same or decreases. However, the failure of router R5 leads to an increase in the lengths of some routes. Histogram on the Figure 14 shows the transmission time only for routes where the broadcast source node is N9. If the source node is another node, the routes will be rebuilt and the histogram will be different.

#### 4.3.4. Calculation of Transmission Route Length in Networks with Tree Topology

Adding redundant routers and cross-links in network with tree topology can help to shorten the broadcast transmission route if the sender and receiver are located in the same tree level. To calculate the length of an alternative route, the length of the original route is divided into two components: the length of the route section that runs between the levels of the tree (Hr) and the length of the route section that runs within one level (Hs):

(9) $$H^{\prime }=Hr+H^{\prime }s$$

where H′ is the length of the original route, reduced by 1 hop (H′ = H − 1), and H′s is the length of the route section that is located within the same level, reduced by 1 hop (H′s = Hs − 1). If the length of the route section that runs within the same level is 0 (i.e., in a situation where the source and receiver of the broadcast are connected to the same switch), H′s = Hs.

The length of the alternative route is calculated by the equation:

(10) $$L=Hr+(N-H^{\prime }s)$$

where N is the number of devices within the same layer (including the redundant router). If the length of the alternative route is less than the length of the original route (L < H′), then broadcasts will be transmitted along the alternative route through the redundant router.

In addition, for networks with tree topology, the length of the broadcast route built when a failure occurs is calculated as follows:

(11) $$Lerr=Hr+(N-H^{\prime }s-2)$$

The worst case is a situation where H′s = 0. This situation can occur if the router that connects the broadcast source and receiver fails. Therefore, the length of the route can increase by N − 2 hops maximum.

In the previously considered example (Figure 13), the maximum route length from node N1 is three hops (to all terminal nodes). As the number of devices in the network increases/decreases, the maximum route length can also increase/decrease.

**Figure 15:**
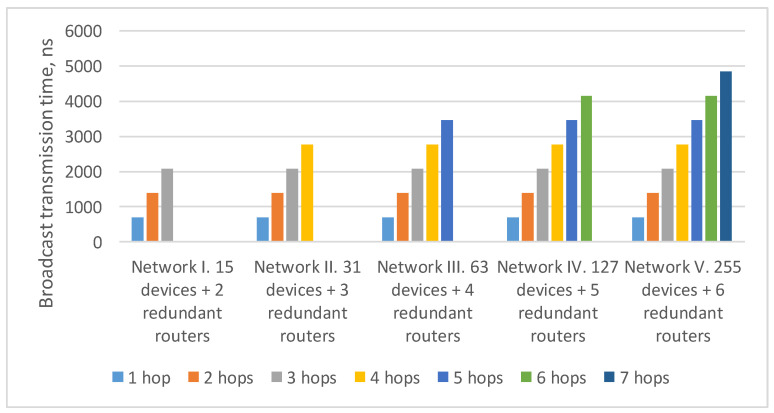
Broadcast maximum transmission time in networks with a tree topology with different route lengths (spatial redundancy with redundant routers).

The Figure 15 shows the broadcast maximum transmission time over networks with a tree topology with different route lengths. It proves that the transmission time for routes of the same length in all networks is equal. At the same time, the maximum transmission time increases linearly depending on the maximum route length.

### 4.4. Comparative Analysis of Spatial Redundancy Methods

**Figure 16:**
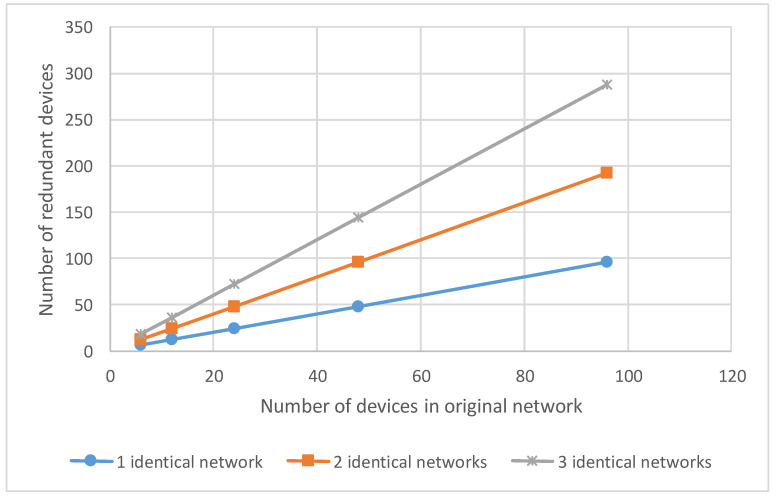
The dependence of the number of additional devices on the number of devices in the network.

The first method of spatial redundancy is based on using identical networks. Regardless of the topology, the number of redundant devices depends on the number of devices in the original network and the number of identical networks, which were added before. The number of redundant devices can be calculated by the equation:

(12) $$N^{\prime }=N\times K$$

where N′ is the number of redundant devices (routers and terminal nodes) in the network after adding K identical networks, N is the number of devices (routers and terminal nodes) in the original network, and K is the number of identical networks. Figure 16 shows a graph of the number of redundant devices versus the number of devices in the original network, using the example of networks with one, two and three identical networks.

Unlike the first method, the second method involves a smaller number of redundant devices. The number of redundant routers that must be connected to the network depends on the network topology.

For 2D-grid topology, the number of redundant routers depends on the number of rows and columns in the original network. The number of redundant routers can be calculated by the equation:

(13) $$R=N_{row}+N_{col}+1$$

where R is the number of redundant routers that must be added in the network, N_row_ is the number of rows in the original network, and N_col_ is the number of columns in the original network. One more redundant router was added to save the regularity of 2D-grid topology. The number of redundant routers does not depend on the number of terminal nodes connected to the routers.

**Figure 17:**
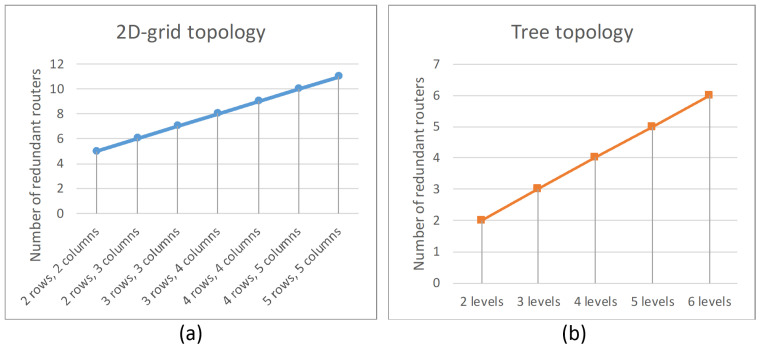
The dependence of the number of additional routers on the: (**a**) number of rows and columns (for 2D-grid topology), (**b**) number of levels of routers (for tree topology).

The Figure 17a shows the number of redundant routers versus the number of rows and columns in the original network.

According to the tree topology, the number of redundant routers depends on the number of levels of routers in the original network. The number of redundant routers can be calculated by the equation:

(14) $$R=N_{lev}$$

where R is the number of redundant routers that must be added in the network, and N_lev_ is the number of levels of routers in the original network. The number of redundant routers does not depend on the number of terminal nodes connected to the routers. In addition, the number of redundant routers does not depend on routers at the same level.

The Figure 17b shows the number of redundant routers versus the number of levels of routers in the original network.

Another advantage of the second spatial redundancy method is the ability to reduce route lengths. When redundant routers are added, cross-links are also added, and switch lines are closed, transforming into a torus, which allows building alternative routes of smaller length.

However, networks that use the second method of spatial redundancy have less fault tolerance compared to networks with redundant routers.

## 5. Analysis of Broadcast Message Propagation over a Network Using Petri Nets

If the number of copies of network structure instances is used to ensure fault tolerance, the various methods of network analysis should be used in order to calculate the value of mitigated failures and the efficiency of broadcast distribution over the network. In such case, the most suitable analysis is the Petri net theory, since it allows analyzing the dynamics of parallel processes and has specific methods of reachability analysis. Petri nets have formal semantics, visual representation and expressiveness. Time Petri nets are used in order to calculate the transmission delay of broadcasts. The difference between Petri nets with time from the classic Petri net is that the transition has an additional time condition. In this case, time is considered not as an absolute value, but as time units, i.e., hours, seconds, nanoseconds, etc. (depending on the process being modeled). Two types of Petri nets can represent them: hard and soft time. For Petri nets with hard time (time Petri nets), the time constraint is the delay λ for firing of a transition (after the transition is allowed). In addition, for a time Petri net, the transition fires during the specified time interval (α, β), where α is the lower and β is the minimum and maximum transition firing time after it becomes allowed [27,28]. For Petri nets with soft time (timed Petri nets), the only time constraint is that the transition fires in a certain time, so each transition is assigned a specific delay value λ only.

To analyze the propagation of broadcast messages, it is sufficient to use timed Petri nets (with soft time). In this case, the delay associated with the transition will consist of the value of the transmission delay on the input channel and the delay in processing the message in the ports, as well as delays inside the device. The calculation of the delay for each transition is represented by the following equation:

(15) $$\lambda (t_{i})=\lambda phy+\lambda port\_in+\lambda port\_out+\lambda n$$

where λphy is the transmission delay on the input channel, λport_in/λport_out is the transmission delay in the input/output port, and λn is the total data-processing latency within the device.

A special rule is proposed to effectively analyze the propagation of broadcasts. This rule represents the propagation of a broadcast message over a network in the form of a Petri net:

1. The Initiator node and a Receiver node of the broadcast message, as well as all switches from the transmission trajectory, are represented as places of the Petri net. The names of specific network devices remain associated with place.
2. The Initiator is marked with one token corresponding to the broadcast message. Thus, the initial marking of the Petri net is μ_0_ = {1, 0, 0, …, 0}.
3. A transition represents the event of transmitting a broadcast message through the corresponding network device. Thus, the channels of the network are transformed into transitions and some arcs of the Petri net connecting the corresponding positions.
4. The multiplicity of arcs cannot be more than 1.
5. The delay for a particular Petri net transition is calculated according to Equation (12).
6. The Receiver can have more than one transition in the input set I(p_i_).
7. The Petri net is built in accordance with the algorithm for constructing a Petri net for broadcast transmission.

An appropriate algorithm was created to build a Petri net that displays broadcast transmission. The algorithm for constructing a Petri net for broadcast transmission is as follows:

1. The algorithm operation starts from the initiator node, marked with one token.
2. Petri net is built:
  - each output channel is transformed into a transition, with one arc entering it with a multiplicity of 1, and the output set of arcs is formed according to the number of channels outgoing from the network device (each can transmit a broadcast);
  - each newly formed arc from the transition is associated with a place corresponding to the network device where the broadcast is transmitted.
  - if the Petri net already has another place corresponding to the same network device, this place is a duplicate (the broadcast has already been delivered to this node), and the construction of this branch of the Petri net stops;
  - move to the next transition, which is not processed by the algorithm;
3. When there are no transitions left for processing in the Petri net, all transitions leading to the receiving node from different network devices are connected into one receiving transition.
4. Building the Petri net is stopped.

**Figure 18:**
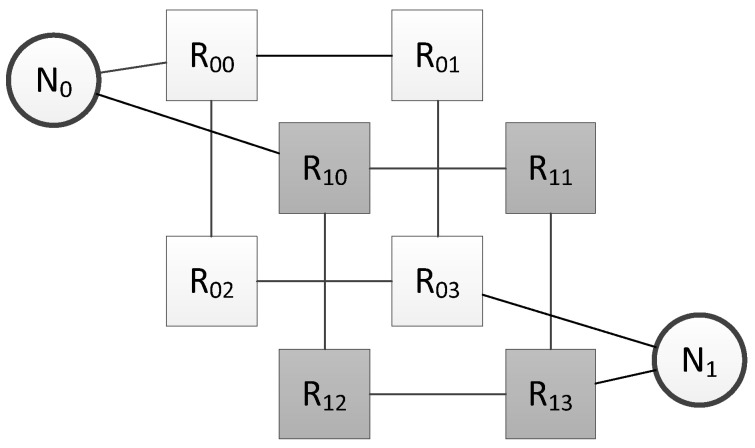
Network topology for Petri net analysis.

Let us consider an example of a network with structural redundancy for which analyzing of the broadcast message distribution is required. The network topology is shown in Figure 18.

**Figure 19:**
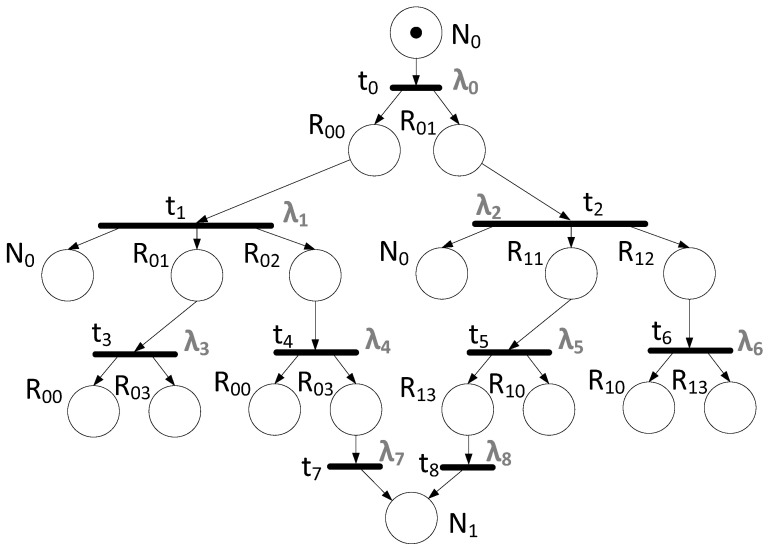
Petri net based on the chosen network topology.

Let us consider the process of transmitting a broadcast message from node N_0_ to node N_1_. The algorithm for constructing a Petri net for broadcast transmission should be applied. The calculation of specific delays corresponding to transitions will not be considered within the framework of this article, since it depends on the specific technical characteristics of the network equipment used. The result of building a Petri net is shown in Figure 19.

A reachability tree does the analysis of the resulting Petri net. Vertices of such a reachability tree are marked with markings that are reached in the process of operation of this net. The root vertex of the reachability tree is marked with the initial marking µ0, and the arcs outgoing from the vertex are marked with all possible transitions t that can be triggered by marking µ_0_ and lead respectively to the vertices marked with directly reachable markings µ_1_, µ_2_, … Further, from each vertex µ_1_, µ_2_, … new arcs come out in accordance with all possible transitions from this marking, etc.

The reachability tree represents all possible transition trigger sequences. Any path in the tree that starts at the root matches a valid hop sequence [29]. This building algorithm is given in detail in [30,31]

**Figure 20:**
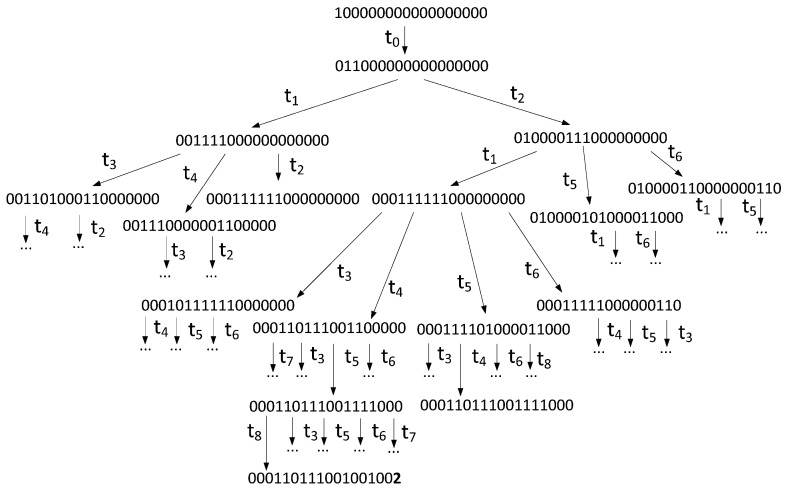
Reachability tree for a Petri net.

The reachability tree built for the Petri net from the Figure 19 is provided in Figure 20. It shows only the main path, since the tree itself is huge.

The resulting tree shows the final marking μ_n_ = {0, 0, 0, 1, 1, 0, 1, 1, 1, 0, 0, 1, 0, 0, 1, 0, 0, 2}, where the number of tokens in place, corresponding to node N_1_, is 2. This means that two broadcast messages can reach the node in the current network. In fact, this proves that the network is resistant to one failure (in case of a second failure, no message will reach the receiver).

This method of analysis also shows that by summing the delays associated with elements from the set of the launch sequence ϭ = {t_0_, t_2_, t_1_, t_4_, t_5_, t_8_}, the delay in transmitting a broadcast message over the network is calculated. It is represented by the following equation:

(16) $$\lambda =\lambda (t_{0})+\lambda (t_{2})+\lambda (t_{1})+\lambda (t_{4})+\lambda (t_{5})+\lambda (t_{8})$$

This method can be used to automate the process of analyzing the propagation of broadcast messages over the network. If the switch breaks down and is not included in the network, the Petri net will not include the corresponding place and the transition associated with it, so the final marking will differ from the one required. To detect this situation, the software implementation of the method (quite simple in terms of code and not resource-intensive) can be periodically launched on the network nodes. Based on the results of the analysis, it is possible to make a decision to reconfigure the network and switch on the cold redundant devices. 

## 6. Broadcast Transmission Parameter Evaluation Using Timed Automata

### 6.1. The Limitations of Previous Methods

Let us consider a small example illustrating the limitations of the previously considered methods for evaluating ongoing processes in dynamics. Consider the case of a network, where at some moment one of the routers fails. If we use the Lie algorithm to evaluate the network characteristics (which was used for the estimation in the first part of the article), the connection graphs (corresponding adjacency or incidence matrices) for two network versions are considered separately. The first version is the one in which the router is present and the second version is the one in which this router is absent. For each of the versions, a reachability tree is formed separately, and the delivery time is estimated. Thus, performance estimation is done for two static states of the system. It is assumed that the failure of the router is equivalent to the absence of this router and the communication lines connected to it in the network. There is no way to estimate what will happen in the system if a router failure occurs directly during the broadcast transmission time period.

It should also be noted that faults and failures could be quite different in their localization and in the forms of manifestation. In relation to the broadcast transmission, the following set of typical failures can be distinguished:

- Inability to receive a broadcast over one of the channels (ports of the router). It occurs as a result of a physical break (rupture) in the communication line, as a result of prolonged noise in the communication line, as a result of failures and faults in the router/terminal node port. In this article, we equate not receiving a broadcast and receiving a broadcast with an incorrect format, incorrect field values, since such a distorted broadcast will be discarded on the receiving side at the Network Layer.
- Inability to transmit the broadcast to one of the channels. The reasons are similar to the previous point. Note that in the case of a break or prolonged noise in the physical channel, both reception and transmission are impossible. Failures and faults inside the port controller can potentially lead to the impossibility of either receiving or transmitting separately.
- Inability to process the broadcast in the network layer. It occurs due to failures and faults in the broadcast controller unit of the network layer or due to the failure of the entire router (for example, power-off).
- Inability to process the broadcast correctly due to the resetting of the router; as a result, information on the history of the broadcast distribution is lost (timeouts, etc.)
- Situations like the “babbling idiot”, in which, because of failures and/or faults, a broadcast with the correct structure spontaneously begins to be sent to the network. Such situations may arise, for example, due to the occurrence of failures of the “stuck-at-1” type for the flags of receiving a broadcast from individual ports, for the flag of the requirement to send a broadcast at the network layer. As a result, such an outwardly correct broadcast can be sent to all or some individual output ports.

Note that failures and faults can occur in the network one by one or in groups. In the second case of several network devices, communication channels go into a failure state simultaneously or almost simultaneously (during a time interval shorter than the time of distribution of the broadcast over the network). Failure of one device can also evolve gradually, whereby the first port may fail, then the second, etc.

If we evaluate the broadcast transmission time using the algorithm described in Section 3 and Section 5, we will need to perform the following actions:

1. identify the stages of the transition process;
2. form connection graphs corresponding to each of the stages of the transition process;
3. perform calculations of characteristics using the algorithm described in Section 3 and Section 5.

At the same time, to determine the stages of the transition process (the study of the transition process itself), to determine the set of these states, another mathematical technique is needed. Timed automata can be used. Note that this technique can also be used to evaluate time characteristics (it can actually implement the algorithm from Section 3 and Section 5).

We propose an approach for the formation of a SpaceFibre network model to determine the set of states that the network passes through in the transient process when failures and faults occur and when the network returns from the states of failures into regular operation and to evaluate the time characteristics.

### 6.2. Proposed Network Model Based on Timed Automata 

As part of this approach, timed automata are used to model network devices (routers and terminal nodes). Broadcasts have the highest priority in relation to other types of information in the SpaceFibre standard, and transmission of other information is temporarily interrupted when a broadcast occurs. Therefore, the presence/absence of other types of data flows in the network does not affect broadcast transmission in any way, despite the fact that the same physical channels between network devices are used for this. As a result, in timed automata, we take into account only the transmission of broadcasts.

The proposed network model has common features with the model proposed for studying scheduling mechanisms in networks [32,33,34]. Similar to this model, we have defined a universal interface for automata—models of network devices. This interface includes channels and global variables.

Routers and terminal nodes used in local networks can potentially have a different number of ports. Therefore, we used an approach based on automata templates, in which the number of ports can be set parametrically. Templates of temporary automata corresponding to routers and terminal nodes were developed to form the network. These templates define the behavior corresponding to the correct operation of devices and operation in case of failures and faults.

**Figure 21:**
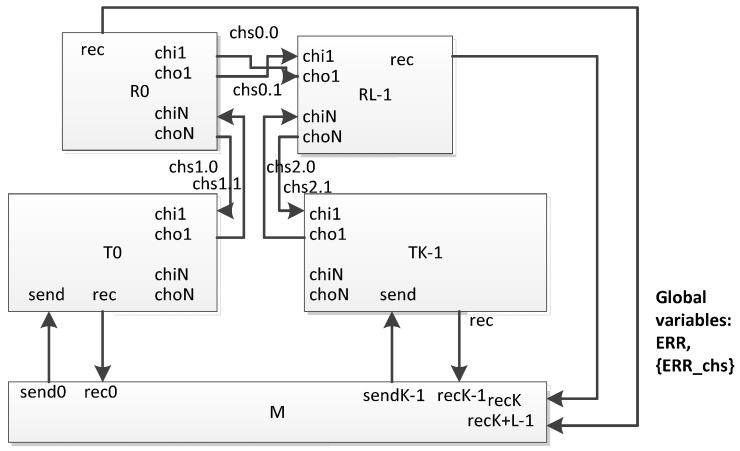
Generalized scheme of a network of timed automata.

To control the process of generating a broadcast by terminal nodes and the time points at which failures and faults should occur in network devices, a special time automaton is used in the network: a simulation process manager. No real device in the network corresponds to this automaton. Such a centralized automaton allows “creating” failures and faults at various time points related to the time of sending the broadcast. This allows us to generate and explore any boundary situations. Also, this automaton is used to estimate the time of distribution of the broadcast over the network. A generalized scheme of the proposed network of timed automata is shown in the Figure 21. It includes three types of automata (in the figure they are represented by rectangles):

- routers (Rj in the figure);
- terminal nodes (Tj);
- the simulation process manager (M).

The following global parameters are defined for the network of timed automata:

- L—the number of routers;
- K—the number of terminal nodes;
- C—the number of communication channels between routers and terminal nodes.

When examining a certain network, the number of routers, terminal nodes and communication channels between them is set in accordance with the graph of connections between network nodes.

For timed automata templates corresponding to routers and terminal nodes, the parameter Nj is defined as the number of external ports of the corresponding network device. Inside the template, for brevity, we will denote this parameter as N. In the automaton model, each port is represented by an input channel chi1 and an output channel cho1, where l ∈ [1, N] (in the SpaceFibre standard, external ports are numbered from 1; the port number 0 is reserved as the internal port of the device used for service applications). These channels are used for communication between routers and terminal nodes. The events transmitted over them correspond to the Broadsasts transmitted in the SpaceFibre network. Global channels, named chsi.j, where i ∈ [0, C − 1], j ∈ [0, 1], are used for communication between the corresponding automata. (Double numbering is used because physical communication channels are bidirectional.) When exploring a network, the channels chsi.j connect time automata, according to the graph of connections for a given network.

The templates of timed automata of terminal nodes interact with the simulation process manager using a set of sendi and reci channels, where i ∈ [0, K − 1]. The send channels are output for the manager’s automaton and input for the terminal node automaton. They transmit events corresponding to the commands for sending a broadcast. Rec channels are output channels for the terminal node automaton and input channels for the manager automaton. The terminal node automaton sends an event to this channel when receiving the broadcast. For many tasks, it is necessary to estimate the time of the broadcast propagation to routers; router templates also interact with the simulation process manager by rec channels.

The dynamic timed automata (DTA) proposed in [35] and the reconfigurable hierarchical timed automata proposed in [36,37] can be used to model the change in network structure that may occur, in particular, due to the occurrence of failures and faults. When using this approach, the structure of connections between the locations of automata can change. Edges can be excluded and added. However, in our work, we used a different approach, based on changing the attributes of transitions between automata locations depending on the values of global variables. Due to this change, the automaton can be used to simulate the correct and incorrect behavior of a network device, as well as its exclusion from the network without changing the structure of connections. This eliminates the need to move from automata with a reconfigurable structure to alternative classical time automata for formal verification.

Two global variables are defined for the network—ERR[L + K] and {ERR_chs}. The variable ERR is an array with Boolean elements. Elements ERR[0] − ERR[L − 1] correspond to routers, elements ERR[L] − ERR[K − 1] correspond to terminal nodes. If ERR[i] = false, then the corresponding automaton functions according to the behavior scheme for a workable device. Otherwise, it functions according to the scheme of behavior for refusal. The values of this variable are controlled by the automaton of the simulation process manager (according to the test scenario). As for the timed automata corresponding to routers and terminal nodes, they are available to read. {ERR_chs} represents a set of global channels for which a failure or faults occurred. The simulation process manager controls the addition of channels to this set and their exclusion from it, according to the test scenario. For the rest of the timed automata, this set is available to read.

The use of these separate variables for modeling failures and faults in routers as a whole and in individual channels allows us to increase the detalization of possible scenarios of system behavior, compared with the approach proposed in [38], in which there is no such gradation.

**Figure 22:**
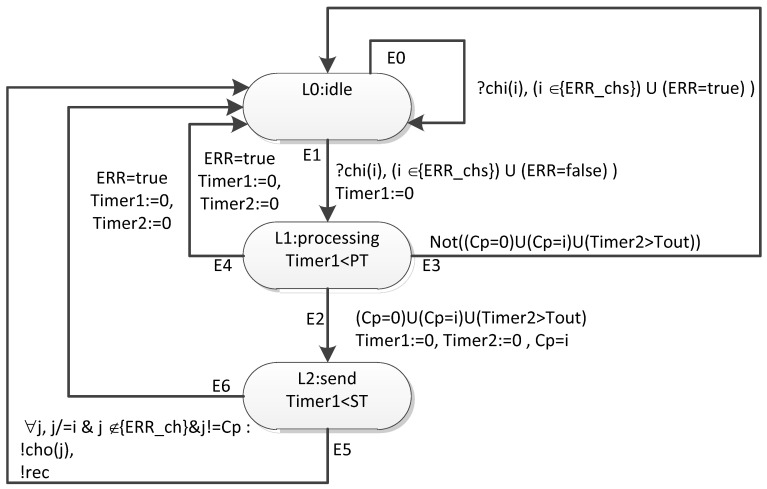
Template of a timed automaton corresponding to the router.

The Figure 22 shows a template of a timed automaton for a router. In this figure, the ovals correspond to locations. A unique identifier and mnemonic designation are specified for each location. For those locations, where the invariant is not a constant “true” value, the invariant is also specified. In this figure, the transitions are indicated by arrows. A unique identifier, transition condition, and related actions are specified for each transition.

In this timed automaton template there are three locations (L0, L1, L2) and six transitions (E0–E5). The template uses two timer variables (Timer1, Timer2). The following parameters are defined for the template:

- PT—the broadcast processing time (λn)
- ST—the broadcast transmission time (λport_out + λphy + λport_in)

A local variable Cp is defined for the template to store the port number from which the broadcast came.

The parameter N is defined for the template as the number of input channels and output channels through which events corresponding to broadcast are transmitted/received. The input channels are indicated by chi(i), and the serial number of the channel is indicated in parentheses. The output channels are indicated by cho(i), with the serial number of the channel again indicated in parentheses.

For the template, the observed variable is ERR (the global variable ERR(i) according to the number of the network device to which this template corresponds). The “true” value corresponds to the state of inability to process the broadcast in the network layer. A global variable is defined for the template—the set {ERR_ch}. It contains the numbers of channels over which the broadcast cannot be transmitted or received. (In this article, to reduce the transition conditions, we have combined these two faults into one.) The values of these variables are controlled by the simulation process manager.

Let us take a closer look at the transition attributes and invariants. The E0 transition is used to simulate failures and faults. If the input channel, through which the next event (broadcast) came, currently belongs to the {ERR_chs} set, the event is discarded without any additional actions. This allows us to simulate situations where there is no possibility of transmission due to a physical disconnection (break) of the line, the effect of long-term noise. If ERR = true, the event is also discarded, which allows us to simulate the current inactivity of this router. (In a real network, in similar situations, the corresponding broadcasts will also be discarded/not sent.) After this transition, the timed automaton returns back to location L0. In general, the automaton can stay in this location for a long time, so the invariant for it has the true value.

The E1 transition is triggered if the event came via a channel that does not currently belong to ERR_chs and ERR = false. This corresponds to the workability of the channel and the router at the current time. The automaton goes to location L1. The Timer1 variable is reset. The automaton will be placed in location L1 either until the time specified by the PT parameter expires (the time of processing the broadcast in the router) or until the ERR variable is set to true (the router has entered an inoperable state). If the automaton is in the L1 location before Timer1 = PT, it goes through the E2 transition if the broadcast is recognized as correct. It also goes through the E3 transition if the broadcast is recognized as incorrect. According to E3, the transition to the L0 location is carried out, which corresponds to the rejection of an incorrect broadcast. If the ERR variable takes the true value when the automaton is in location L1, it goes to location L0 by transition E4, which corresponds to the failure of the router.

The automaton can be in the L2 location either until ERR=true, or until Timer1 is less than the ST parameter. (The value of this parameter determines the time when the broadcast is sent via channels.) If ERR = true, then the automaton goes to location L0, which corresponds to the failure of the router. If the automaton performs an E5 transition, then events corresponding to broadcast are sent to all workable output channels, except the one from which it came. The automaton goes to location L0.

Thus, transitions E0, E4, E6 provide the possibility of modeling failures and faults of routers and communication channels during network operation.

**Figure 23:**
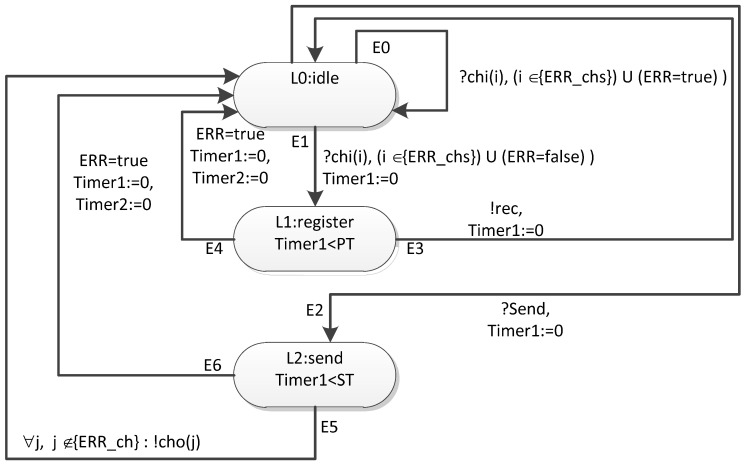
Template of a timed automaton corresponding to a terminal node.

Figure 23 shows a timed automaton template for a terminal node.

Unlike the router model, the terminal node model registers received broadcast, but does not send events corresponding to broadcast distribution to the output channels. An event is sent to the simulation process manager via the rec channel.

Sending an event corresponding to the broadcast is carried out by a command from the simulation process manager (the send channel is used for this).

On the basis of such timed automata templates, networks with an arbitrary topology (any graph of connections between routers and terminal nodes) can be formed.

The SystemC library version 2.1 was used to implement and simulate a network of timed automata, but later versions can also be used. The templates of the automata were implemented based on SC_HAS_PROCESS. Channels between automata were implemented on the basis of SC_SIGNAL. Using SystemC allows us to implement additional functionality required for dynamic reconfiguration. These features are difficult to implement when using tools for studying timed automata networks such as UPPAAL [39].

**Figure 24:**
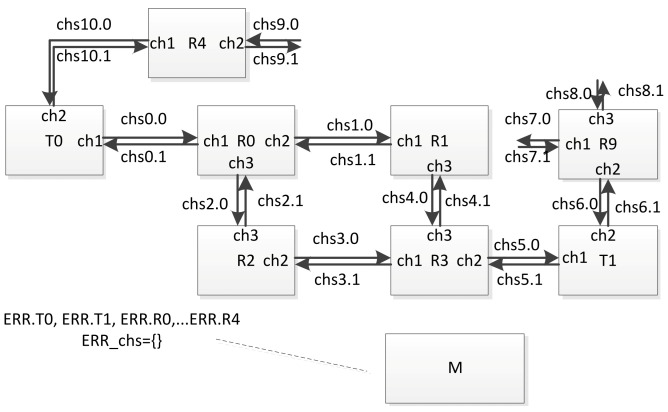
An example of a fragment of a timed automata network.

An example of a fragment of a network is shown in the Figure 24.

**Figure 25:**
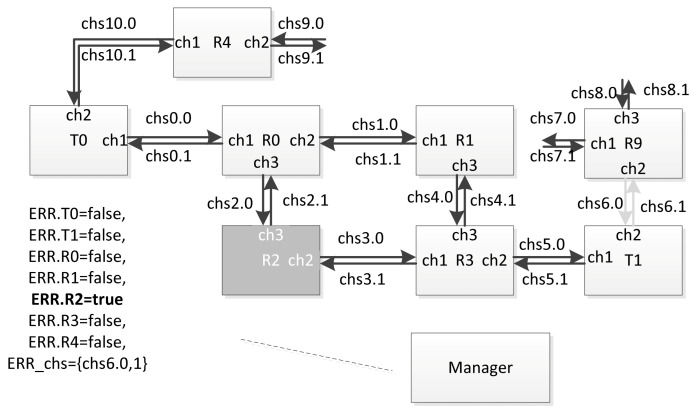
An example of the network configuration with the router and communication channel disconnected.

The variable ERR.Ti is assigned to each terminal node, and the variable ERR.Ri is assigned to each router (from the ERR array). In order to simulate a failure or fault of a network node occurring at some point in time, the simulation process manager sets the corresponding variable to ‘true’. In case of failure simulation, after some time, this variable can be set to ‘false’ again. In order to simulate a failure or fault of a communication channel, the identifier of the corresponding channel is placed in the set ERR_chs. If a failure simulation is performed, then after a while, the ID of this channel can be excluded from this set. For example, at some point in time, the simulation process manager can assign the variable ERR.R2 = true, ERR_chs = {chs6}. As a result, the current network configuration will look as shown in the Figure 25.

In this figure, the components that are considered inoperable in the current configuration are marked in light gray. If the timed automaton corresponding to router R2 was not in location L0 at the time of the configuration change, it will move to location L0. If events (corresponding to broadcasts) are sent to it via channels chs2 and chs3, they will be discarded. Events will not be sent to channel ch2 (global identifier chs6) in the automaton corresponding to router R9. Similarly, in the automaton corresponding to terminal node T1, events will not be sent to channel ch2 (global identifier chs6).

The timed automaton is the manager of the simulation process functions according to the test scenarios. It changes the values of ERR and {ERR_chs}, sends events to sendi channels, receives events from reci channels. For the test manager, L + K timers Timer_ti, i ∈ [0, L + K − 1] are defined to estimate the time of the broadcast propagation to terminal nodes and routers. These timers are reset when the event is sent via the sendi channel. The values of the corresponding timers at the time of receiving events over the reci channel allow us to determine the delivery time to the corresponding network devices.

Optional variables can be added to the network of timed automata, which allow collecting various statistical information during the modeling process. In particular, a broadcast_counter variable can be added for each channel, which allows estimating the number of broadcasts in channels, because the actual part of the channel bandwidth is occupied by broadcast transmission. The broadcast counter action should be added to the set of actions related to the E5 transition (broadcast_counter = broadcast_counter + 1;) for the router and the terminal node automata templates to implement this evaluation.

### 6.3. Example of Proposed Approach Use

**Figure 26:**
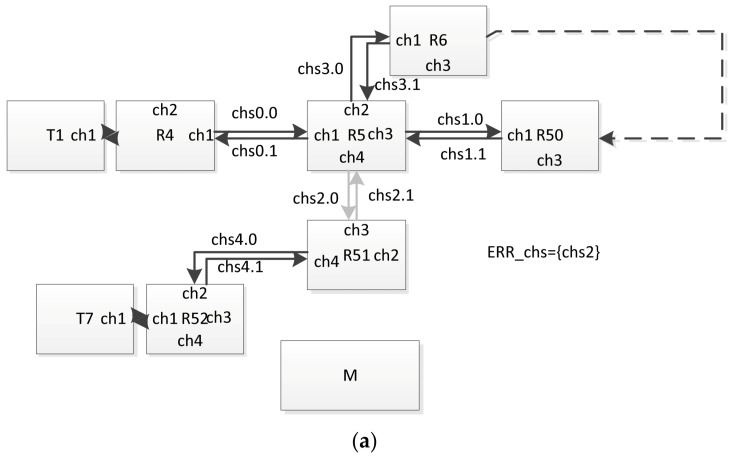

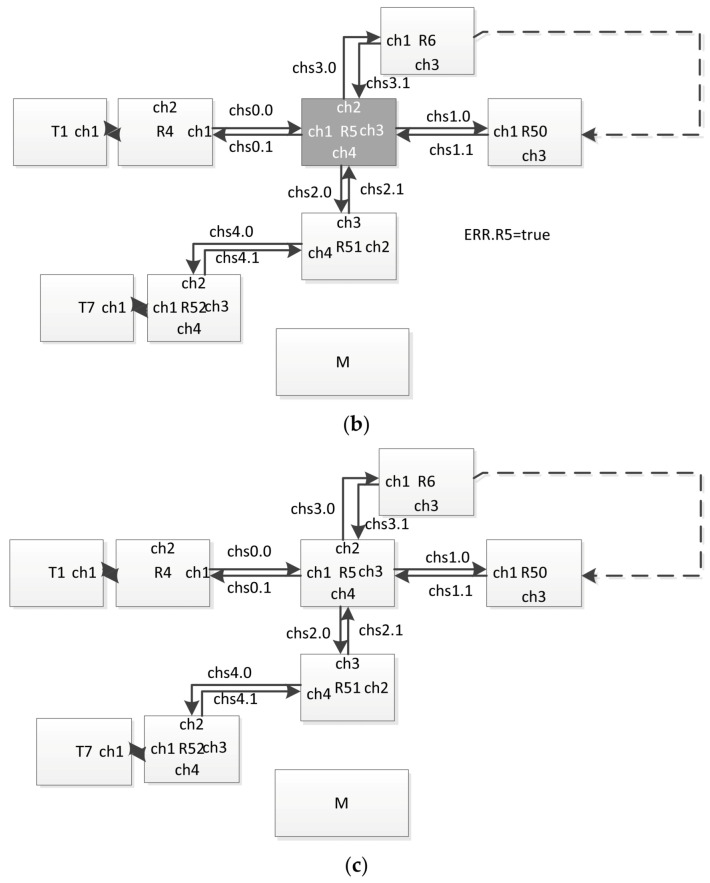
Example of a network fragment: (**a**) the channel ch4 (chi4, cho4) of the router R5 is in a malfunction state, (**b**) entire router R5 is in reset state, (**c**) the router R5 and all its channels operate correctly.

The use of this approach, in particular, allows us to obtain a more accurate estimation of the broadcast propagation time in the presence of failures in the network. To illustrate this, we give the following example of a network fragment, shown in Figure 26. In this example, the shortest path of the broadcast propagation is between T1 and T7:

T1 → R4 → R5 → R51 → R52 → T7.

The time of the broadcast distribution can be determined for it using the method proposed in Section 3 and Section 5.

The following system behavior scenario was considered. At the time of passing the considered broadcast through router R5, its channel ch4 (chi4, cho4) was in a state of malfunction (Figure 26a). Immediately after passing the broadcast via this router, the variable ERR was set to true (Figure 26b). Then, after a time less than the time of the broadcast propagation along path R6–R50, this variable was set to false (Figure 26c). (This corresponds to the restart of the router.) Next, this broadcast came via channel 3 to the router and was sent through channels 1, 2 and 4. As a result, this broadcast reached the R51 router via the following path:

T1 → R4 → R5 → R6 → … → R50 → R5 → R51 → R52 → T7.

For transmission along this path, it took significantly longer time than that which can be calculated when building a distribution tree without taking into account such a possible failure–recovery scheme. (This broadcast also reached routers R4, R6 and R50, but if no reset was performed, it is defined as a doublet). Potentially, such a situation can lead to an incorrect interpretation of this broadcast (a sequence of broadcasts) in terminal nodes connected to router R51. This problem may occur when, for example, another broadcast (a broadcast corresponding to another event with a different channel number, but logically related to the one being checked) is sent over the network after a sufficient time for the distribution of the first broadcast in the absence of failures.

## 7. Conclusions

The article discusses the dependency of the broadcast maximum transmission time of used methods of spatial redundancy. Examples of using spatial redundancy methods on two-dimensional grids and tree topologies are considered.

The use of spatial redundancy methods allows to mitigate faults that occur along the path of broadcast maximum transmission time over the network. The lengths of the built routes are in many cases less than or equal to the lengths of the original routes, or exceed them by a small number of hops.

Also, the addition of redundant routers and cross-links allows us to shorten broadcast maximum transmission routes when the network is operating correctly without failures.

The introduced algorithm for Petri net construction, which displays the transmission of a broadcast message, makes it possible to automate the process of assessing network fault tolerance, as well as to estimate the delay of broadcast transmission to recipient nodes. This algorithm can be extremely useful for speeding up the process of fault mitigation and reconfiguring the network in real time.

The article also proposes an approach to evaluate the characteristics of broadcast transmission in the SpaceFibre network based on timed automata and suggests templates of timed automata that allow evaluating characteristics in conditions of dynamically occurring failures and faults. This allows studying the process of distribution of broadcasts in the SpaceFibre network in conditions of possible failures and faults in detail. Failures and faults can be modeled with a high level of detail. In particular, it is possible to evaluate the consequences of failures and faults of individual router ports (especially sequential degradation of the router). The rest of the router remains functioning correctly. As a result, accurate evaluation of the characteristics of the system becomes possible under various scenarios of failures and faults.

## Data Availability

Data sharing is not applicable to this article.

## References

[1] (2019). SpaceFibre—Very high-Speed Serial Link.

[2] Alur R., Dill D. (1994). A theory of timed automata. Theor. Comp. Sci..

[3] Karpov Y.G. (2010). Model Checking. Verification of Parallel and Distributed Software Systems.

[4] Abd Alrahman Y., Azzopardi S., Piterman N., Margaria T., Steffen B. (2022). Model Checking Reconfigurable Interacting Systems. Leveraging Applications of Formal Methods, Verification and Validation. Adaptation and Learning.

[5] Velder S.E., Lukin M.A., Shalyto A.A., Yaminov B.R. (2011). Verification of Automata-Based Programs.

[6] Govind R., Herbreteau F., Srivathsan B., Walukiewicz I. Abstractions for the local-time semantics of timed automata: A foundation for partial-order methods. Proceedings of the 37th Annual ACM/IEEE Symposium on Logic in Computer Science.

[7] Alur R., La Torre S., Pappas G. (2004). Optimal Paths in Weighted Timed Automata. Theor. Comp. Sci..

[8] André E., Lime D., Roux O.H., Ogata K., Lawford M., Liu S. (2016). Decision Problems for Parametric Timed Automata. ICFEM: Formal Methods and Software Engineering. Lecture Notes in Computer Science.

[9] André E., Lime D., Ramparison M., Jansen D.N., Prabhakar P. (2018). TCTL model checking lower/upper-bound parametric timed automata without invariants. FORMATS: Formal Modeling and Analysis of Timed Systems. Lecture Notes in Computer Science.

[10] Jensen P.G., Kiviriga A., Guldstrand Larsen K., Nyman U., Mijaika A., Høiriis Mortensen J., Ábrahám E.E., Paolieri M. (2022). Monte Carlo Tree Search for Priced Timed Automata. Quantitative Evaluation of Systems, Proceedings of the 19th International Conference (QEST 2022), Warsaw, Poland, 13–16 September 2022.

[11] Beneš N., Bezděk P., Larsen K.G., Srba J. (2015). Language Emptiness of Continuous-Time Parametric Timed Automata. Part II. Automata, Languages, and Programming, Lecture Notes in Computer Science, Proceedings of the 42nd International Colloquium, ICALP 2015, Kyoto, Japan, 6–10 July 2015.

[12] André E. (2019). What’s decidable about parametric timed automata?. Int. J. Softw. Tools Technol. Transf..

[13] Chakraborty S., Phan L.T.H., Thiagarajan P.S. Event Count Automata: A State-based Model for Stream Processing Systems. Proceedings of the 26th IEEE International Real-Time Systems Symposium.

[14] Boyer M., Roux P. Embedding network calculus and event stream theory in a common model. Proceedings of the 21st International Conference on Emerging Technologies and Factory Automation (ETFA).

[15] Hammal Y., Monnet Q., Mokdad L., Ben-Othman J., Abdelli A. (2014). Timed automata based modeling and verification of denial of service attacks in wireless sensor networks. Stud. Inform. Univ..

[16] Anand M., Dajani-Brown S., Vestal S., Lee I. Formal Modeling and Analysis of AFDX Frame Management Design. Proceedings of the 9th International Symposium on Object-Oriented Real-Time Distributed Computing.

[17] Govind R., Herbreteau F., Srivathsan B., Walukiewicz I. Revisiting local time semantics for networks of timed automata. Proceedings of the 30th International Conference on Concurrency Theory.

[18] Akshay S., Gastin P., Govind R., Srivathsan B., Klin B., Lasota S., Anca Muscholl A. (2022). Simulations for Event-Clock Automata. Proceedings of the 33rd International Conference on Concurrency Theory.

[19] Zhan Y., Hsiao M.S., Baldoni M., Bandini S. (2021). Breaking Down High-Level Robot Path-Finding Abstractions in Natural Language Programming. Advances in Artificial Intelligence.

[20] Sherwani N. (1998). Algorithms for VLSI Physical Design Automation.

[21] Nannipieri P., Fanucci L., Siegle F. (2020). A representative SpaceFibre network evaluation: Features, performances and future trends. Acta Astronaut..

[22] Baillieul J., Antsaklis P.J. (2007). Control and Communication Challenges in Networked Real-Time Systems. Proc. IEEE.

[23] Shooman M.L. (2002). Reliability of Computer Systems and Networks: Fault Tolerance, Analysis, and Design.

[24] Alena R.L., Ossenfort J.P., Laws K.I., Goforth A. Communications for Integrated Modular Avionics. Proceedings of the IEEE Conference on Aerospace.

[25] Butz H. (2007). The airbus approach to open integrated modular avionics (IMA): Technology, methods, processes and future road map. Aircraft Systems Technician.

[26] Aeronautical Radio Inc. (2005). Aircraft Data Network Part 7: Avionics Full-Duplex Switched Ethernet (AFDX).

[27] Huang B., Zhou M., Lu X.S., Abusorrah A. (2023). Scheduling of Resource Allocation Systems with Timed Petri Nets: A Survey. ACM Comp. Surv..

[28] Komenda J., Zorzenon D., Balun J. (2022). Modeling of safe timed Petri nets by two-level (max,+) automata. IFAC.

[29] Olenev V. (2022). A methodology for formalized development of communication protocols based on Petri nets. Inf. Space.

[30] Dixon A., Lazić R., Biere A., Parker D. (2020). KReach: A Tool for Reachability in Petri Nets. Tools and Algorithms for the Construction and Analysis of Systems, Proceedings of the 28th International Conference, Munich, Germany, 2–7 April 2022.

[31] Liu G. (2022). Petri Nets: Theoretical Models and Analysis Methods for Concurrent Systems.

[32] Glonina A.B., Balashov V.V. (2018). On the Correctness of Real-Time Modular Computer Systems Modeling with Stopwatch Automata Networks. Model. Anal. Inf. Syst..

[33] Glonina A.B. (2020). A Tool System for Schedulability Analysis of Modular Computer Systems Configurations.

[34] Glonina A.B. (2018). General model of real-time modular computer systems operation for checking acceptability of such systems configurations. Bulletin of the South Ural State University, Series: Mathematical Modelling, Programming and Computer Software.

[35] Tigane S., Guerrouf F., Hamani N., Kahloul L., Khalgui M., Ali M.A. (2023). Dynamic Timed Automata for Reconfigurable System Modeling and Verification. Axioms.

[36] Bettira R., Kahloul L., Khalgui M., Li Z. Reconfigurable Hierarchical Timed Automata: Modeling and Stochastic Verification. Proceedings of the 2019 IEEE International Conference on Systems, Man, and Cybernetics.

[37] Bettira R., Kahloul L., Khalgui M. A Novel Approach for Repairing Reconfigurable Hierarchical Timed Automata. Proceedings of the 15th International Conference on Evaluation of Novel Approaches to Software Engineering.

[38] Tahiri I., Philippot A., Carre-Menetrier V., Tajer A. TimeBased Estimator for Control Reconfiguration of Discrete Event Systems (DES). Proceedings of the CoDIT 2019: International Conference on Control, Decision and Information Technologies.

[39] UPPAAL Online Documentation. https://uppaal.org/documentation.

